# Metachronous metastasis to inguinal lymph nodes from sigmoid colon adenocarcinoma with abdominal wall metastasis: a case report

**DOI:** 10.1186/s12885-019-5386-x

**Published:** 2019-02-27

**Authors:** Taro Tanabe, Dai Shida, Shunsuke Tsukamoto, Goki Morizono, Hirokazu Taniguchi, Yukihide Kanemitsu

**Affiliations:** 1 Department of Colorectal Surgery, National Cancer Center Hospital, 5-1-1, Tsukiji, Chuo-ku, Tokyo, 1040045 Japan; 2 Department of Pathology and Clinical Laboratories, National Cancer Center Hospital, 5-1-1, Tsukiji, Chuo-ku, Tokyo, 1040045 Japan

**Keywords:** Colon cancer, Inguinal lymph node metastasis, Abdominal wall metastasis

## Abstract

**Background:**

Inguinal lymph node metastasis from rectum is uncommon but well-known occurrence, whereas that from colon adenocarcinoma is extremely rare. Inguinal lymph node metastasis from colon adenocarcinoma has only been reported in previous cases involving primary tumor invasion of the abdominal wall, or in those involving colon cancer metastasis to external iliac lymph nodes. We describe a case of inguinal lymph node metastasis from colon cancer without primary tumor invasion to the abdominal wall.

**Case presentation:**

A 42-year-old female, who had undergone twice cesarean sections before, underwent open sigmoidectomy for sigmoid colon adenocarcinoma and received 12 cycles of FOLFOX regimen as adjuvant chemotherapy. Two years after sigmoidectomy, a follow-up CT scan revealed enlarged inguinal lymph nodes as well as growth of enhanced mass lesions on the abdominal wall at site of the cesarean section scar. Biopsy of both lesions revealed well-differentiated adenocarcinoma, and immunohistochemistry demonstrated positive expression of CDX2, substantiating its gastrointestinal origin. We therefore performed dissection of left inguinal lymph nodes and mass lesion of the abdominal wall. The patient died 51 months after lymph node dissection.

**Conclusions:**

This is the first reported case of inguinal lymph node metastasis from colon cancer without invasion of the primary tumor to the abdominal wall or without involvement of the external iliac lymph nodes, suggesting that the pathway of inguinal metastasis originated from the abdominal wall metastasis. When inguinal lymph node metastasis from colon cancer is suspected, if an R0 resection was possible, inguinal lymph node dissection may be a potentially effective treatment.

**Electronic supplementary material:**

The online version of this article (10.1186/s12885-019-5386-x) contains supplementary material, which is available to authorized users.

## Background

Inguinal lymph node metastasis from lower rectum is uncommon but well-known occurrence [1], whereas that from colon adenocarcinoma is extremely rare. The reason for its rarity is that lymphatic vessels of the colon usually run along the superior or inferior mesenteric arteries, rather than along the external iliac or femoral artery system, the latter of which is thought to represent the primary route for inguinal lymph node metastasis from lower rectal tumors [2].

A literature search of reports published in English identified only five cases of colon adenocarcinoma with inguinal lymph node metastasis [3–7]. In four of these cases, the primary tumor was suspected to invading the abdominal wall or skin, and was then thought to have metastasized to inguinal lymph nodes through the superficial lymphatic pathway along the inferior epigastric artery [3–5, 7]. In the remaining case, ascending colon cancer metastasized to external iliac and inguinal lymph nodes, suggesting that inguinal lymph node metastasis had occurred through the external iliac system [6]. In this report, we describe a case of metachronous metastasis to inguinal lymph nodes from a sigmoid colon adenocarcinoma, with neither abdominal wall invasion of the primary tumor nor involvement of the external iliac lymph nodes, but with synchronous abdominal wall metastasis.

## Case presentation

**Fig. 1:**
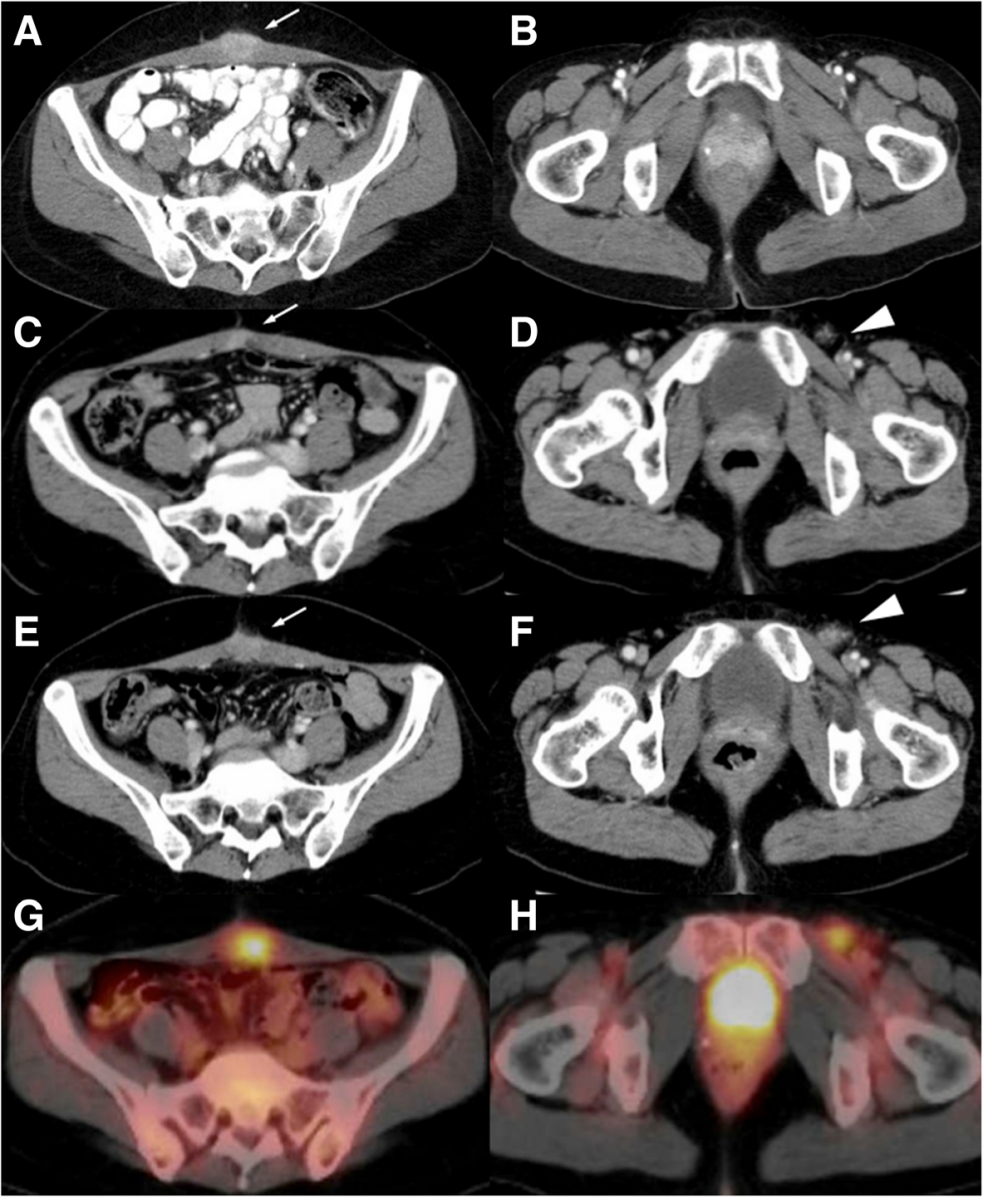
**a**-**b**. CT scan before colectomy shows an enhanced mass lesion in abdominal wall (arrow). No enlarged inguinal lymph nodes are evident. **c**-**d**. After adjuvant chemotherapy, the mass lesion in abdominal wall decreased in size (arrow). **e**-**f**. Two years post-colectomy, CT shows regrowth of a mass lesion (arrow) and enlarged left inguinal lymph nodes (arrow head). **g**-**h**. Two years post-colectomy, PET-CT shows significant accumulation of 18F-fluorodeoxy glucose in both lesions

**Fig. 2:**
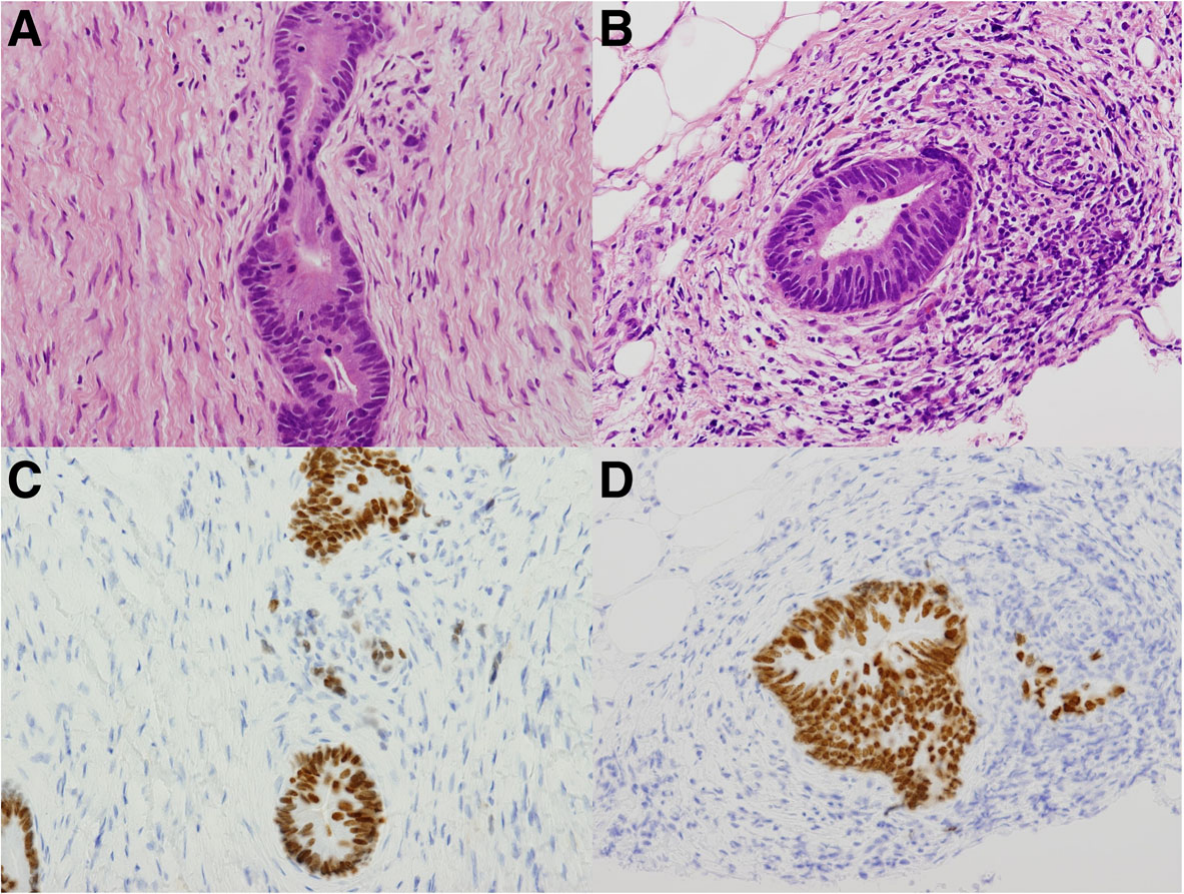
**a**-**b**. Histopathological findings suggest that both the abdominal mass lesion and left inguinal lymph node indicate well-differentiated adenocarcinoma (H&E staining). **c**-**d**. Immunohistochemical examination revealed that both lesions were positive for CDX2, substantiating the gastrointestinal origin

A 42-year-old woman who had undergone twice cesarean sections presented with hematochezia and was evaluated by CT scan in August 2011. A CT only detected an enhanced abdominal wall mass near the abdominal scar, which was thought to be desmoid at the time (Fig. 1a and b). As the hematochezia continued, a further endoscopic workup was performed, and well-differentiated adenocarcinoma was detected at sigmoid colon. The whole body CT scan detected no other distant metastasis. She underwent elective open sigmoidectomy with D3 lymph node dissection for sigmoid colon cancer with mid-line incision in May 2012. Histology of the sigmoid colon showed well-differentiated adenocarcinoma invading to subserosa with 8 out of 20 regional lymph nodes involved. The sigmoid colon cancer was classified as pT3N2b, according to the TNM classification of the Union for International Cancer Control Eights Edition [8]. Molecular analysis showed KRAS mutation and microsatellite-stable. Postoperative course after sigmoidectomy was uneventful and the patient was treated with 12 cycles of FOLFOX regimen as adjuvant chemotherapy. The patient entered a scheduled clinical follow up program which included: regular physical examinations and CEA test every 3 months, and whole-body CT every 6 months. CT after adjuvant chemotherapy revealed the lesion on the abdominal wall had decreased in size and inguinal lymph nodes were all normal in size (Fig. 1c and d). In April 2014, a routine follow-up CT scan revealed enlarged left inguinal lymph nodes as well as a growing enhanced mass lesion on the abdominal wall at the site of cesarean section scar (Fig. 1e and f). 18F-fluorodeoxy glucose positron emission tomography (FDG-PET) revealed significant accumulation at both lesions (Fig. 1g and h). A CT showed no distant metastases were detected in other organs and laboratory data showed serum CEA level was within the normal range (1.3 ng/ml). Although metastases to both inguinal lymph nodes and the abdominal wall from colon adenocarcinoma are clinically uncommon, needle biopsy of those lesions was performed with the suspicion of inguinal lymph node recurrence and abdominal wall metastasis from colon cancer. Histopathological findings indicated well-differentiated adenocarcinoma, and immunohistochemistry revealed positive expression of CDX-2, substantiating its gastrointestinal origin (Fig. 2). Thus, under the diagnosis of sigmoid colon cancer recurrence in left inguinal lymph nodes and synchronous abdominal wall metastasis, because abdominal wall mass was exist when primary tumor resection, we performed left inguinal lymph node dissection and resection of the abdominal wall with reconstruction using an anterolateral thigh flap, with a curative intent. Intraoperative findings showed no evidence of dissemination, distant metastasis, or other non-curative clinical factors. Pathology revealed well to moderately differentiated adenocarcinoma in both lesions. Three of the eight retrieved inguinal lymph nodes showed involvement of adenocarcinoma. The patient had a favorable postoperative course and was discharged from the hospital without any complications.

Follow-up CT one year after the inguinal lymph node dissection showed multiple distant lymph node metastases, including some in para-aortic lymph nodes. The patient received FOLFIRI as palliative chemotherapy, but died due to disease progression 51 months after inguinal lymph node dissection (Additional file 1).

## Discussion and conclusions

We reported a rare case of metachronous inguinal lymph node metastasis from colon adenocarcinoma with simultaneous metastasis to the abdominal wall. To date, only five cases of colon cancer patients with inguinal lymph node metastasis who underwent curative resection have been reported (Table 1). Previous reports have claimed that possible mechanism of metastasizing from colon cancer to inguinal lymph nodes is that tumor invading to the abdominal wall metastasize to the inguinal lymph nodes through superficial lymphatic pathways along the inferior epigastric artery. Different from previous reports [3–7], our case was not confirmed tumor invasion to the abdominal wall but may have synchronous abdominal wall metastasis. It can be presumed that inguinal lymph node metastasis seems to originate from the abdominal wall metastasis, which seems to be the first case to date.

**Table 1:** Review of reported cases of colon adenocarcinoma with inguinal lymph node metastases

Author	Age	Gender	Location	Differentiation	pStage	Synchronous or metachronous	Other places of metastases	Survival after inguinal lymph node dissection
Hakeem A, et al. [3]	60	Female	Cecum	Moderately differentiated	T3N2M1	Synchronous	none	Not described
Pisanu A, et al. [7]	62	Male	Sigmoid colon	Not described	T4bN1M0	Metachronous	none	6 months, alive without recurrence
Hara M, et al. [4]	67	Male	Cecum	Mucinous	T4bN1M0	Metachronous	none	36 months, alive without recurrence
Iwamoto M, et al. [5]	82	Female	Sigmoid colon	Moderately differentiated	T4bN1M1	Synchronous	none	60 months, alive without recurrence
Kitano Y, et al. [6]	83	Female	Ascending colon	Well-differentiated	T3N0M1	Synchronous	none	27 months, alive without recurrence
Current case	42	Female	Sigmoid colon	Well-differentiated	T3N2M1	Metachronous	Abdominal wall	51 months, died due to disease progression

Whereas metastasis to skeletal muscle, such as abdominal wall, from colon adenocarcinoma is rare [9], injury to skeletal muscle may alter muscle physiology, rendering it more susceptible to metastatic disease at such sites [10]. Our patient had undergone twice cesarean sections before primary tumor resection, and metastasis to skeletal muscle occurred at the previously documented sites of skeletal muscle trauma. Thus, muscle trauma from previous surgeries may be associated with synchronous abdominal wall metastasis and remaining abdominal wall metastasis may lead to inguinal lymph node metastasis in this case.

Although non-regional lymph node metastases are generally considered to spread systemically [11], 4 cases of colon cancer with inguinal lymph node metastasis reportedly showed no recurrence after lymph node dissection [4–7]. In our case, the patient lived more than four years after inguinal lymph node dissection despite of peritoneal dissemination recurrence one year after inguinal lymph node dissection. This result suggests that surgical treatment for inguinal lymph node metastases from colon cancer might improve prognosis in select cases. Further observation of a larger population of such patients is required to clarify proper and effective treatments for such cases, including the dissection of distant lymph node metastasis.

We report the first case of metachronous inguinal lymph node metastasis from sigmoid colon adenocarcinoma with synchronous abdominal wall metastasis. Our findings suggest that metastasis to inguinal lymph nodes followed a pathway that began with abdominal wall metastasis. Inguinal lymph node dissection for the inguinal lymph node metastasis from colon cancer may be an effective treatment if an R0 resection can be achieved.

## Additional file

Additional file 1: Timeline of the case. Time line: The “timeline” visualizes the course of treatment of the patient from the time of first detection of the tumor to the time of last follow-up. (DOCX 150 kb)

## Abbreviations

CDX-2: Caudal-type homeobox protein 2
CEA: carcinoembryo antigen
CT: computed tomography
FOLFIRI: Folinic acid; 5-fluorouracil; irinotecan
FOLFOX: Folinic acid; 5-fluorouracil; oxaliplatin
PET: positron emission tomography
