# Supplementary material for: Metachronous metastasis to inguinal lymph nodes from sigmoid colon adenocarcinoma with abdominal wall metastasis: a case report
Source: BMC Cancer. 2019 Feb 27;19:180. doi: 10.1186/s12885-019-5386-x (PMC6391797; doi:10.1186/s12885-019-5386-x)
Supplement: Supplementary file 1 — Timeline of the case. Time line: The “timeline” visualizes the course of treatment of the patient from the time of first detection of the tumor to the time of last follow-up. (DOCX 150 kb) [file 12885_2019_5386_MOESM1_ESM.docx]

Timeline

42 years old female with past medical history of twice cesarean section

Cesarean section

2010

2009

8/2011

Patient was still alive 3.5 years after inguinal lymph node dissection

CT:

Peritoneal dissemination

Therapeutic chemotherapy

FOLFIRI

CT:

Multiple lymph node metastases

8/2015

Left inguinal lymph node dissection

Resection of the abdominal wall with reconstruction

Diagnosis:

Inguinal lymph node metastasis

Abdominal wall metastasis

CT:

Enlarged inguinal lymph nodes

Growing abdominal mass lesion

Biopsy of left inguinal lymph node and abdominal wall mass

Diagnosis:

Sigmoid colon cancer

pT3N2bM0 StageIIIC

Neo adjuvant chemotherapy

FOLFOX 12 cycles

Open Sigmoidectomy

5/2012

Hematochezia

4/2014

7/2014
